# Interoception across Modalities: On the Relationship between Cardiac Awareness and the Sensitivity for Gastric Functions

**DOI:** 10.1371/journal.pone.0036646

**Published:** 2012-05-11

**Authors:** Beate M. Herbert, Eric R. Muth, Olga Pollatos, Cornelia Herbert

**Affiliations:** 1 Department of Psychosomatic Medicine and Psychotherapy, University Hospital Tuebingen, Eberhard-Karls-University Tuebingen, Tuebingen, Germany; 2 Department of Psychology, Clemson University, Clemson, South Carolina, United States of America; 3 Department of Psychology, Psychology of Motivation and Emotion, University of Potsdam, Potsdam, Germany; 4 Department of Psychology I, Biological Psychology, Clinical Psychology and Psychotherapy, University of Wuerzburg, Wuerzburg, Germany; Royal Holloway, University of London, United Kingdom

## Abstract

The individual sensitivity for ones internal bodily signals (“interoceptive awareness”) has been shown to be of relevance for a broad range of cognitive and affective functions. Interoceptive awareness has been primarily assessed via measuring the sensitivity for ones cardiac signals (“cardiac awareness”) which can be non-invasively measured by heartbeat perception tasks. It is an open question whether cardiac awareness is related to the sensitivity for other bodily, visceral functions. This study investigated the relationship between cardiac awareness and the sensitivity for gastric functions in healthy female persons by using non-invasive methods. Heartbeat perception as a measure for cardiac awareness was assessed by a heartbeat tracking task and gastric sensitivity was assessed by a water load test. Gastric myoelectrical activity was measured by electrogastrography (EGG) and subjective feelings of fullness, valence, arousal and nausea were assessed. The results show that cardiac awareness was inversely correlated with ingested water volume and with normogastric activity after water load. However, persons with good and poor cardiac awareness did not differ in their subjective ratings of fullness, nausea and affective feelings after drinking. This suggests that good heartbeat perceivers ingested less water because they subjectively felt more intense signals of fullness during this lower amount of water intake compared to poor heartbeat perceivers who ingested more water until feeling the same signs of fullness. These findings demonstrate that cardiac awareness is related to greater sensitivity for gastric functions, suggesting that there is a general sensitivity for interoceptive processes across the gastric and cardiac modality.

## Introduction

The perception and feedback of visceral signals is considered to be an essential variable in many theories of emotion [1]–[4], suggesting a close relationship between the perception and processing of bodily signals (“interoception”) and emotional experience and cognition. Additionally, it has been shown, that it is not the mere occurrence of physiological changes but their perception and a person’s individual ability to perceive visceral responses (“interoceptive awareness”) that affects emotional, cognitive and behavioral functions [5]–[10], with benefits in decision-making [11] and attentive processes [12], and behavioral self-regulation [13].

Attempts to quantify the individual degree of accurately perceiving one’s internal bodily signals have predominantly focused on the individual sensitivity for cardiac signals (“cardiac awareness”) that has been assumed to represent an indicator of general “interoceptive awareness”. Investigating cardiac awareness is primarily based on the advantage that the sensitivity for cardiac signals can be easily and non-invasively measured by heartbeat perception tasks [14]–[16]. Typically, two kinds of heartbeat perception tasks have been used and have been shown to be well validated and reliable (Cronbach’s α.69-.90) [17]–[18]: “Heartbeat tracking or detection tasks” ask participants to count their heartbeat silently for brief time periods without feeling for their pulse [15]. In “heartbeat discrimination tasks” participants are required to determine whether heartbeat sensations occur simultaneously with external stimuli (e.g., tones, lights, vibrations), which are presented at different time delays following the onset of the R-wave of the electrocardiogram (ECG) [16], [19].

Although cardiac awareness can be reliably detected, the implicit assumption of cardiac awareness representing an indicator of “general” interoceptive awareness for bodily signals is still unresolved. It is not clear whether the individual sensitivity for cardiac activity is indeed related to the sensitivity for other bodily, visceral functions that would justify speaking of an individual general interoceptive awareness across different visceral bodily modalities.

Anatomical studies demonstrated a class of afferent fibers that monitor the physiological state of all internal organs of the body [20], which converge to “interoceptive centers” in the insular cortex [20]–[22] and give rise to conscious visceral perception, i.e. interoception. It has been stated that interoception represents the sense of the physiological condition of the entire body and of different bodily and visceral inputs including the processing and the perception of a broad range of somatic and visceral signals and feeling [21]–[22]. In support of this, there is ample evidence that many different physiological responses arising from different visceral systems activate the anterior insula including thirst, dyspnea, air hunger, the Valsalva manoeuvre, sensual touch, pain, itch, penile stimulation, sexual arousal, coolness, warmth, exercise, heartbeat, distension of the bladder, stomach, rectum or oesophagus [22]–[27]. Additionally, regarding cardiac awareness, both heartbeat perception tasks used to estimate cardiac awareness have been shown to involve the anterior insula [6], [28]–[30] and good compared to poor heartbeat perceivers show greater activation especially in the anterior insula [6], [30], indicating better central representation and integration of cardiovascular signals in persons with good cardiac awareness.

According to this evidence and because there are quite stable inter-individual, trait-like differences in perceiving ones cardiac signals [8], [10], [13], [15], [18], [30], it might be proposed that interoceptive awareness involves a general sensitivity for bodily, visceral signals across different visceral tissues.

Studies investigating interoception across different visceral modalities in healthy persons under controlled conditions are very sparse and findings are ambivalent [31]. This is particularly due to the fact that measuring the sensitivity for visceral bodily functions is limited by the existing possibilities of how to specifically stimulate a selected organ system and/or how to reliably assess and to individually perceive spontaneous activity of a bodily system. Furthermore, some bodily signals, such as heartbeats, are more easily to perceive than other visceral signals [32] and there is evidence that in most arousing situations there is only a small correspondence of physiological reactivity in different organ systems [33]. These aspects can obviously hamper finding “coherence” in the detection or awareness of different bodily signals and might explain some findings suggesting a lack of congruency of perceptions of different interoceptive signals such as heart rate, breathing or sweating hands within the same person [31].

One bodily system that can be manipulated and measured using standardized methods and that shows spontaneous activity which can be perceived comparably to cardiac activity under controlled conditions is the gastrointestinal (GI) system. Although there has been longstanding interest in interoceptive processes in the GI tract [23]–[24], however, unlike studies on interoception in the cardiovascular system, which have investigated mainly healthy subjects, studies on the GI tract have focused considerably on functional gut disorders by using invasive methods of distension of the rectum, stomach or oesophagus [24], [27], [34]–[39]. To date there is only one study that investigated awareness of both cardiac and gastrointestinal sensations in healthy persons under experimentally controlled conditions. Whitehead and Drescher [16] examined 20 healthy students and showed that perceptual sensitivity scores of gastric and cardiac perception were correlated significantly (r = .51) with each other, suggesting a generalized tendency to be aware of visceral events. However, these results are based on invasive assessments. Stomach contractions were assessed by the perfused catheter method where sterile water was perfused constantly past the sensing element of a physiological pressure transducer and into the stomach through a nasogastric tube. Stomach contractions were measured as pressure changes and participants were asked to indicate whether a light coincided with a felt contraction. Heartbeat perception was assessed by a discrimination method.

Using invasive methods of assessing gastric sensitivity has several limitations which may potentially influence the relevant dependent functions. Using this method implies that participants should tolerate the procedure and are taught to swallow a stomach tube during preparatory sessions before the experiment [16]. This requires a pre-selection of participants for these studies. In addition, the training of swallowing a gastric tube already represents an iteratively applied gastric stimulus that is potentially able to induce learning effects of gastric sensations, thus sensitizing the participants in perceiving their gastric functions. Moreover, it has been criticized that invasively applied stimuli in order to stimulate gastric functions are to a great degree artificial and lack ecological validity [40]. Furthermore, this study used a heartbeat discrimination method and a comparable simultaneity paradigm of detecting the invasively stimulated gastric signals, and it is unresolved if results hold true for assessing cardiac awareness by using a heartbeat tracking task and investigating gastric sensitivity by non-invasive procedures that are not biased by limitations of invasive measurements.

Thus, the principal aim of the present study was to investigate the relationship between cardiac awareness as assessed by a standard heartbeat tracking task [15] and the sensitivity for gastric functions by the use of non-invasive methods of measuring gastric perception and gastric activity in a larger sample of healthy individuals.

For this purpose we employed a standardized water load test (WLT) representing an easily performed, well tolerated and reliable method to induce gastric distension and postingestion gastric neuromuscular activity, in order to assess gastric sensation [34]–[36], [41]. The drink test has been demonstrated to show high reproducibility in healthy subjects as well as in patients with functional gastrointestinal disorders [35]–[36], [42], correlates well with invasive, barostat methods used to induce gastric distensions [43]–[44] and maximal ingested water volume in the WLT has been demonstrated to represent a valid indicator for subjectively felt fullness [35]–[36], [41]. Comparable to results on invasive balloon-distension suggesting visceral hypersensitivity in patients with functional GI disorders [27], [37], it has been shown that e.g. patients with functional dyspepsia or irritable bowel syndrome ingest less volumes of water in the WLT compared to healthy controls due to supposed greater visceral sensitivity and more sensitive perception of fullness from gastric distension [34]–[36], [45].

In healthy persons water loads have been demonstrated to stimulate normal slow-wave frequency of the stomach in humans which is 3 cpm (cycles per minute) [36], [41], [46]. Gastric myoelectrical activity can be non-invasively assessed via the electrogastrogram (EGG) that has been shown to represent a reliable and valid technique to record gastric activity that regulates gastric motility [36], [46]–[47]. This increase in normogastria is also observed after eating, sham feeding or when expecting to eat pleasant food [46], [48]–[51], whereas gastric dysrhythmias (bradygastria and tachygastria) are associated with nausea and vomiting, nausea of motion sickness and symptoms of nausea in diverse groups of patients with gastric disorders [35]–[36], [42], [46], [52]–[54].

On the basis of this evidence showing that the WLT is a sensitive marker for gastric sensitivity, and proposing that interoceptive awareness implies interoception across different bodily systems, we hypothesized that cardiac awareness should be accompanied by less water ingestion in the WLT until feeling first signs of fullness, signaling more sensitive perception of gastric function. In accordance with earlier findings [36], [41], [46], we also hypothesized that different amounts of water loads were associated with different 3 cpm activity of the stomach as measured by the EGG.

## Materials and Methods

### Participants

Sixty-eight right-handed female students were recruited by notices posted on the campus of the University of Tuebingen. All interested women filled in screening questionnaires regarding sociodemographic and health information. Items included age, educational status, body weight and body height, current and former illnesses (e.g. eating disorders and/or disordered eating behavior, psychiatric diseases, cardiac diseases, gastrointestinal diseases, diseases of the respiratory system, diabetes, and further internal and metabolic diseases, infections, craniocerebral injuries, accidents, etc.), medication use, and sporting activities. Questions on gastrointestinal symptoms also included low-level conditions such as heartburn, dyspepsia, bloating or irritable bowel syndrome (IBS).

Only healthy female participants without any diseases, without use of medication and without substance abuse were selected for inclusion in the study. As it is known that regular exercise influences autonomic tone, especially the vagal component [55]–[56], which in turn is able to improve cardiac awareness as assessed by heartbeat perception [8], [57]–[58], only individuals not regularly involved in athletic or endurance sports participated in this study.

We aimed at avoiding sex differences and therefore investigated only a sample of women because it has been shown that men potentially demonstrate more accurate heartbeat perception than women [24], [59] and this relationship seems to be mediated by body mass [18], [60]. Furthermore, men have been demonstrated to potentially ingest greater water volumes during the WLT than women [35], [61]. Additionally, there are data reporting that body mass index (BMI) and overweight is positively related to reduced satiety [62] and fasting gastric volumes [62]–[63], although there are equivocal findings [63]–[64]. To ensure that these variables did not influence our results only women with a normal range of the BMI (ranging from 19-25) were chosen for participation.

Based on these criteria nineteen students had to be excluded. The final sample comprised 49 healthy female students with an age range of 21 to 45 years (*M* = 25.19; *SD* = 4.30) and a body mass index (BMI) of *M* = 21.35 (*SD* = 1.99). None of these final participants had received out- or inpatient treatment for somatic diseases, mental disorders or eating disorders and none suffered from cardiovascular or gastrointestinal diseases. There was no medication use and all participants had an equivalent educational background and were students. All participants gave written informed consent and all experiments were conducted in accordance with the declaration of Helsinki. Ethical approval was obtained from the Institutional Review Board of the University Clinic of Tuebingen.

### Procedure

All subjects were studied in the morning after 4 hours of fasting. In preparation for the study, all subjects were asked not to consume any alcohol or caffeine or take any medication the day before the study. After arrival at the laboratory participants rated their subjective feelings of hunger and thirst via a self-report scale ranging from 0: not hungry/thirsty at all to 10: very hungry/thirsty.

Following the exclusion of participants based on the screening questionnaire, the remaining participants were led into a sound-attenuated room and were fitted with non-polarizable Ag-AgCl adhesive disposable electrodes for electrocardiogram (ECG) and electrogastrogram (EGG) recording and filled in the State Trait Anxiety Inventory (STAI) [65]. All recordings were performed in a quiet room with the participant instructed not to talk and to remain as still as possible during recording to minimize motion artifacts. Then, baseline measurements of ECG, EGG and respiration were recorded for 15 minutes. Respiration was assessed using a flexible strain gauge that was placed on the chest or abdomen depending on body type in order to avoid interfering with the EGG electrodes. Subsequently, all participants completed the heartbeat perception task and after a pause the WLT. After completion of the WLT, EGG and respiration recordings were continued for an additional 30 minutes [35], [41]. All measurements were done in half-supine position of the participants.

### Cardiac Awareness

Cardiac awareness was assessed using a heartbeat tracking task [7]–[8], [13], [15], [30]. The heartbeat tracking task was performed according to the mental tracking method by Schandry [15] using four intervals of 25, 35, 45, and 55 seconds that were separated by standard resting periods (30 seconds). During all trials ECG was recorded and participants were requested to concentrate on their heart activity and count their own heartbeats. A start and stop cue signaled the beginning and the end of the counting phases. Participants were not permitted to take their pulse or to attempt any other manipulations that could facilitate the detection of heartbeats. Following the stop signals, participants were asked to verbally report the number of counted heartbeats. The participants were not informed about the length of the counting phase nor about the quality of their performance.

A heartbeat perception score was calculated as the mean score across four heartbeat perception intervals according to the following transformation: ¼ Σ (1– (|recorded heartbeats – counted heartbeats|)/recorded heartbeats). The heartbeat perception score varies between 0 and 1. The maximum score of 1 indicates absolute accuracy of heartbeat perception.

Furthermore, the sample was divided into good (good HP) and poor heartbeat perceivers (poor HP) according to the heartbeat perception score. Participants scoring above 0.85 were assigned to the good heartbeat perception group (N = 20), whereas the remainder formed the group of poor heartbeat perceivers (N = 29). The selected cut-off score of 0.85 was used in accordance with many previous studies [7], [12]–[13], [30], [66]–[67], which showed that this score is appropriate for distinguishing between individuals who substantially differ in cardiac awareness.

### Water Load Test (WLT)

WLT was performed by having subjects drink room temperature, non-carbonated water ad libitum over a 5-min period until reaching the point of individually perceived fullness [34]–[36]. Participants were instructed to stop drinking when they felt first signs of fullness. Water was consumed from an unmarked flask that was taken from the subject and refilled after each drink. The volume required to refill the flask to the initial level was recorded, and the total volume consumed was calculated by summing these volumes. In this way, the flask was “bottomless” and the participants were blinded as to the actual volume of water consumed. Directly after the test participants rated symptoms of fullness/satiety, nausea, felt valence (pleasantness and unpleasantness) and arousal during the water ingestion procedure on a self-report scale ranging from 0: no sensation/not at all to 10: very severe.

### Electrogastrogram (EGG) and Electrocardiogram (ECG)

After skin preparation, three standard Ag-AgCl electrodes (Cleartrace, Conmed, Utica, NY USA) for EGG were positioned on the anterior abdominal surface. All electrode sites were cleaned with abrasive gel and wiped with a gauze pad prior to electrode placement. Electrogastrogram (EGG) data were assessed using the UFI 3991 x/3 GPP BioLog (UFI Corp., Morro Bay, CA) and EGG data were collected at a sampling rate of 10 Hz. Respiration was used for cross referencing with the EGG in order to identify and eliminate data with respiration artifacts. EGG recordings were visually inspected for strong respiratory artifacts that could make the EGG data unusable. This occurs when the EGG picks up respiration instead of the electrical activity of the stomach. This can occur when the diaphragm is in close proximity to the stomach and the respiratory cycle moves the stomach in a way that the EGG records respiration instead of the electrical activity. Periods deemed to have strong respiratory artifacts were excluded from analysis. This was not necessary in any of the participants.

The EGG signal was analyzed using a Fast-Fourier running spectral analysis [46], [68]–[69]. A Hamming window was applied to 2048 points of data and successive windows were overlapped by 75%. Spectral estimates from the multiple windows were averaged separately for each subject and each condition to result in a single series of estimates. Total power was calculated as a sum of the spectral estimates from 1–10 cycles per minute (cpm). Percentage of total power was calculated for the bradygastria or bradyarrhythmia (1–2.5 cpm), normogastria 3 cpm (2.5–3.5 cpm) and tachyarrhythmia or tachygastria (3.75–10 cpm) bands.

ECG was recorded by a computer-based data acquisition system and the corresponding software (Task Force Monitor 3040i, Ver. 2.001, CNSystems Medizintechnik GmbH, Austria). ECG was measured by means of lead II, Einthoven, with a sampling rate of 1000 Hz. Heart rate data were used for assessing the heartbeat perception score.

### Questionnaire Data

Since former data suggested a positive association between cardiac awareness and anxiety [58], [70], the participants filled in the German version of the State Trait Anxiety Inventory (STAI) [71] at the beginning of the examinations. The STAI is a 40 item scale, which assesses both state and trait anxiety and represents well-validated and reliable self-report measures of dispositional and state anxiety. The scales for trait and state anxiety are made up of 20 items each to which respondents are asked to indicate to what degree the items describes their dispositional and situational feelings on a four-point Likert-type scale (where 1 =  “not at all” and 4 =  “very much so”).

### Data Analysis

Changes in EGG activity from 15 minutes baseline to reactivity during/after the WLT (5 minutes WLT procedure plus 30 minutes after WLT) were analyzed by means of repeated measures analyses of variance (ANOVAs) with the within-subjects factor “water ingestion” (activity during baseline vs. activity during WLT) for each EGG frequency band.

Pearson correlations were calculated between the heartbeat perception score and the amount of water volume (ml) each participant ingested until feeling full in order to investigate the relationship between heartbeat perception sensitivity and the sensitivity for gastric functions. Additionally, Pearson correlations were analyzed between the heartbeat perception score and the percentages of total power for bradygastria (%bradygastria), normogastria (%3 cpm), and tachyarrhythmia (%tachyarrhythmia) frequency bands during baseline and during and after 30 minutes of WLT. Furthermore, bivariate Pearson correlations were calculated between ingested water volume (ml) and % 3 cpm activity during the WLT. In order to analyze if the association between heartbeat perception score and %3 cpm activity during the WLT was mediated by the ingested water volume (ml), ingested water volume (ml) was included as a predictor in linear multiple regression analyses with %3 cpm activity as the criterion and heartbeat perception score as a predictor.

Correlations were also examined between the heartbeat perception score and subjective ratings of fullness, pleasant and unpleasant feelings and nausea as well as between the water load (ml) and recorded feelings of hunger and thirst at the beginning of the study.

Differences between good and poor heartbeat perceivers regarding ingested water volume (ml) and subjective ratings of perceived fullness, nausea, valence and arousal during the WLT procedure were analyzed by means of ANOVAs with “group” (good vs. poor heartbeat perceivers) as between-subjects factor. Differences between both groups in baseline EGG activity and EGG reactivity scores to the WLT procedure in all frequency bands (%3 cpm, %bradyarrhythmia, %tachyarrhythmia) were analyzed by using ANOVAs with “group” as between-subjects factor. Where appropriate (when the assumption of sphericity was not met), degrees of freedom were adjusted according to Greenhouse and Geisser [72]. Uncorrected F values are reported together with unadjusted degrees of freedom and adjusted p-values. For evaluation of significant (p<.05) main effects, critical differences were determined using the Scheffé procedure.

Finally, Pearson correlations were analyzed between state and trait anxiety scores and heartbeat perception scores. Possible group differences in anxiety scores were analyzed by use of ANOVAs.

## Results

### Gastric Activity (EGG) During Baseline and WLT

**Figure 1:**
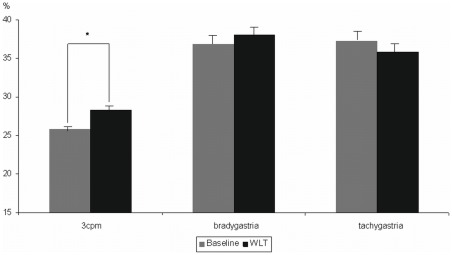
Activity of the electrogastrogram (%3 cpm, %bradygastria, %tachygastria) during baseline and during/after water load test (*: p<.05); total power was calculated as a sum of the spectral estimates from 1–10 cpm, percentage of total power was calculated for the bradygastria (1–2.5 cpm)  =  %bradygastria, normogastria 3 cpm (2.5–3.5 cpm)  =  %3 **cpm, and tachygastria (3.75–10 cpm) bands  =  %tachygastria.**

ANOVA results demonstrated that %3 cpm activity increased from baseline to 30 minutes after the WLT, *F*(1,48) = 4.83, *p* = .03, *η^2^* = .09. There were no significant changes in %bradygastria, *F*(1,48) = 0.70, *p = *.45, *η^2^ = *.01 and in %tachyarrhythmia, *F*(1,48) = 0.90, *p* = .34, *η^2^* = .02 (see Figure 1).

### Heartbeat Perception, Ingested Water Volume and EGG Activity

Mean heartbeat perception score for all participants (N = 49) was *M* = 0.70, *SD* = 0.17 and ranged from 0.24 to 0.99. This distribution of the heartbeat perception score is comparable to the distribution found in earlier studies in healthy participants [8], [30].

**Figure 2:**
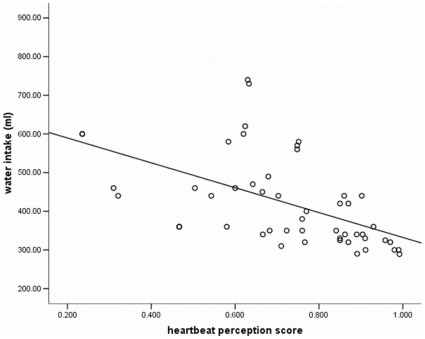
Correlation plot between heartbeat perception score and ingested water volume (ml) (r = −.50, p = .001).

**Figure 3:**
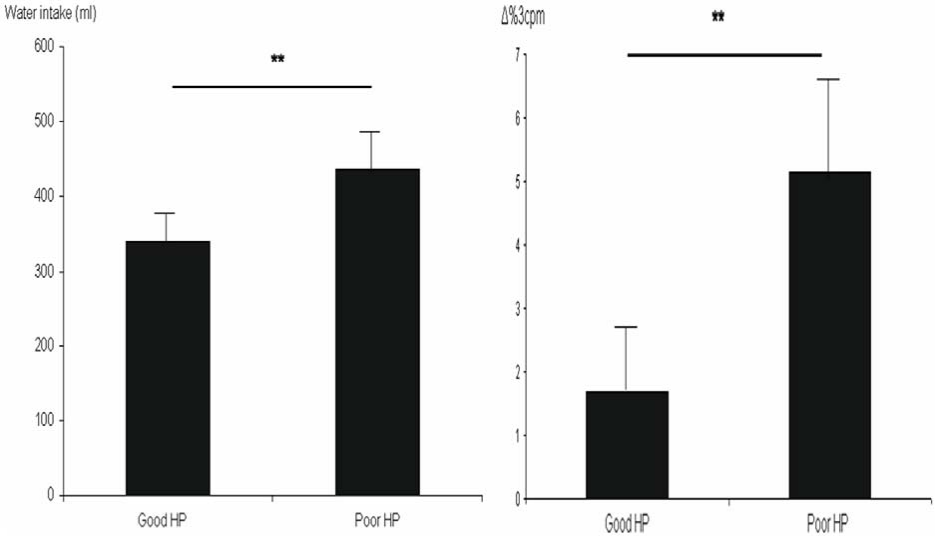
Water intake (ml) in good and poor heartbeat perceivers (**: p<.01), good HP  =  good heartbeat perceivers, poor HP  =  poor heartbeat perceivers (3A); Normogastric reactivity (% 3 cpm) in good and poor heartbeat perceivers during/after WLT (**: p<.01); % 3 **cpm is percentage of the 3 cpm activity (2.5-3.5 cpm) relative to the total power (3B).**

There was a significant negative correlation between the heartbeat perception scores and the amount of ingested water volume (*r* = −.50, *p* = .001) (see Figure 2). In accordance with these results, ANOVA results demonstrated that good compared to poor heartbeat perceivers ingested significantly less water (good HP: N = 20, *M* = 349.67 ml, *SD* = 44.19 ml; poor HP: N = 29, *M* = 458.81, *SD* = 108.63 ml), *F*(1,45) = 16.59, *p* = .005, *η^2^* = .23 (see Figure 3A).

Additionally, there was a significant negative correlation between heartbeat perception scores and %3 cpm EGG activity during and 30 minutes after the WLT procedure (*r* = −.29, *p* = .04) showing that cardiac awareness was associated with less %3 cpm activity during the WLT procedure. There were no significant correlations between %3 cpm and heartbeat perception score during baseline (*r* = −.09, *p* = .52) and between %bradygastria (*r* = .09, *p* = .54) or %tachygastria (*r* = −.13, *p* = .34) during/after WLT or during baseline (%bradygastria: r = .18, p = .21; %tachygastria: *r* = −.12, *p* = .20) and heartbeat perception scores.

There was also a significant correlation between %3 cpm activity and ingested water volume (ml) in the WLT (r = .37, p = .03). In accordance with the results of the bivariate correlation analyses the results of the regression analyses demonstrated that the heartbeat perception score significantly predicted %3 cpm activity during the WLT (β = −.29, t = −2.03, R^2^ = .08, p = .04) and that the heartbeat perception score significantly predicted ingested water volume (ml) (β = −.50, t = −4.09, R^2^ = .25, p = .001). Additionally, ingested water volume (ml) significantly predicted %3 cpm activity (β = .37, t = 2.10, R^2^ = .14, p = .03).

When entering ingested water volume (ml) in addition to the heartbeat perception score as predictors into the linear multiple regression analyses with %3 cpm activity as criterion the results demonstrated that ingested water volume (ml) continued to significantly predict %3 cpm (β = .35, t = 1.99, R^2^ = .12, p = .03) while the heartbeat perception score no longer significantly predicted %3 cpm activity (β = −.13, t = −.09, R^2^ = .02, p = .21).

ANOVA analyses for group differences regarding EGG reactivity demonstrated that good heartbeat perceivers (*M* = 1.70, *SD* = 7.81) showed significantly less %3 cpm increase during WLT than poor heartbeat perceivers (*M* = 5.15, *SD* = 7.50), *F*(1,48) = 6.95, *p* = .01, *η^2^* = .13) (see Figure 3B). There were no significant differences between good and poor heartbeat perceivers for baseline EGG activity: %3 cpm (good HP: *M* = 26.64, *SD* = 5.33, poor HP: *M* = 25.82, *SD* = 6.24), *F*(1,48) = .14, *p* = .71, *η^2^* = .003*;* %bradygastria (good HP: *M* = 37.60, *SD* = 5.96, poor HP: *M* = 36.51, *SD* = 9.17), *F*(1,48) = .13, *p* = .73, *η^2^* = .003; %tachygastria (good HP: *M* = 35.76, *SD* = 8.81, poor HP: *M* = 37.79, *SD* = 8.43), *F*(1,48) = .46, *p* = .50, *η^2^* = .01.

### Subjective Ratings and Anxiety

ANOVAs demonstrated no significant differences between good and poor heartbeat perceivers in the subjective ratings of fullness (good HP: *M* = 3.17, *SD* = 3.85, poor HP: *M* = 3.36, *SD* = 2.82, *F*(1,48) = ,002, *p* = .96, η^2^ = .001), arousal (good HP: *M* = 0.91, *SD* = 1.92, poor HP: *M* = 1.15, *SD* = 1.61, *F*(1,48) = .67, *p* = .67, *η^2^* = .004), pleasant feelings (good HP: *M* = 8.15, *SD* = 1.28, poor HP: *M* = 7.72, *SD* = 1.71, *F*(1,48) = 1.52, *p* = .22, *η^2^* = .03), unpleasant feelings (good HP: *M* = 1.08, *SD* = 1.31, poor HP: *M* = 1.50, *SD* = 1.81, *F*(1,48) = 2.65, *p* = .21, *η^2^* = .04) and nausea (good HP: *M* = 0.10, *SD* = .29, poor HP: *M* = .36, *SD* = 0.99, *F*(1,48) = .87, *p* = .35, *η^2^* = .02).

Correlation analyses demonstrated that there was no significant association between state (r = .04, p = .39) or trait anxiety (*r* = .09, *p* = .36) and heartbeat perception score as well as no significant correlation between heartbeat perception score and subjective ratings of fullness (r = .04, p = .40), arousal (r = .06, p = .38), pleasant feelings (r = .09, p = .29), unpleasant feelings (r = .08, p = .30) and nausea (r = .09, p = .31).

There were no significant differences between good (trait anxiety: *M* = 35.23, *SD* = 7.64; state anxiety: *M* = 35.80, *SD* = 4.86) and poor heartbeat perceivers (trait anxiety: *M* = 37.41, *SD* = 8.97; state anxiety: *M* = 36.45, *SD* = 8.52; trait anxiety: *F*(1,48) = .62, *p* = .43, *η^2^* = .01; state anxiety: *F*(1,48) = .08, *p* = .78, *η^2^* = .001). Additionally, there was no significant correlation between the volume of ingested water in the WLT and state anxiety (*r* = −.10, *p* = .45) or trait anxiety (*r* = .04, *p* = .79) as well as no significant correlation between ingested water (ml) and subjective feelings of hunger (M = 4.67, SD = 0.98; r = .03, p = .81) and thirst (M = 4.89, SD = 1.12; r = .04, p = .80) at the beginning of the study.

## Discussion

The present study investigated the relationship between cardiac awareness using a heartbeat tracking task and the sensitivity for gastric functions assessed by means of a water load test (WLT), in association with gastric myoelectrical activity as measured by non-invasive electrogastrogram (EGG) in healthy persons. The results demonstrate that there is a significant negative association between cardiac awareness and the amount of water ingested ad libitum that represents an indicator of subjectively felt fullness [35]–[36], [41]. Accordingly, persons with habitually good cardiac awareness showed significantly less water intake until experiencing first signs of fullness compared to persons with poor cardiac awareness. These results suggest that the sensitivity for cardiac signals is associated with the sensitivity for gastric feedback as can be subjectively perceived as feeling signs of fullness of the stomach. Our findings confirm and extend earlier results of a study by Whitehead and Drescher [16] that proposed a general sensitivity for interoceptive processes regarding both cardiac and gastric signals.

The results of the present study extend this earlier finding with respect to several relevant aspects. Whereas Whitehead and Drescher used a heartbeat discrimination task that is based on simultaneity paradigms, this study investigated cardiac awareness by means of a heartbeat tracking task. Thus, the findings of both studies confirm a positive association between cardiac awareness and the sensitivity for gastric signals that is independent of the applied heartbeat perception technique. An important aspect of the present study is that non-invasive methods of measuring gastric sensitivity and functions were used in order to assess interoceptive awareness across both, cardiac and gastric, organ systems. As has been stated before, the earlier study applied invasive methods to measure gastric perception which implies potential relevant limitations regarding a pre-selection of appropriate and compliant study participants for these assessments as well as a potential bias of sensitization for gastric signals by repetitively practicing and getting used to the swallowing of gastric tubes. Furthermore, the invasively applied distension stimuli are to a great degree artificial [40] and are not directly comparable to a method such as heartbeat perception which can be measured without invasive manipulation of organ functions. The present study used a non-invasive method of a drink test that induces gastric distension and evokes gastric motility responses without the complex hormonal response of a caloric test meal and with ingested water volume representing a valid index of feeling full. This allows assessing an estimate of the sensitivity for gastric signals [34]–[36], [41]. Thus, our results, confirm coherence of interoception across two relevant bodily visceral modalities.

This is underscored by both, observed changes in gastric activity and subjective experience during water load. As expected, the EGG results demonstrated that ingestion and post-ingestion of drinking water in the whole group resulted in a significant increase of the percentage of normogastria (%3 cpm), but no significant changes in bradygastria and tachyarrhythmia. This represents a typical and normal gastric reaction after water load in healthy humans [35], [46]. Additionally, cardiac awareness was significantly associated with a smaller increase of normogastria (%3 cpm) during the ingestion and post-ingestion period of water intake and, accordingly, good compared to poor heartbeat perceivers demonstrated significantly less increase of %3 cpm during the WLT procedure. However, there were no significant associations between heartbeat perception scores and subjective ratings of fullness/satiety or nausea and felt valence or arousal after the WLT, or any differences between good and poor heartbeat perceivers in these ratings.

These findings suggest that persons with good cardiac awareness indeed ingested less water because they subjectively felt more intense signals of fullness during this lower amount of water intake compared to persons with poor cardiac awareness who ingested more water until feeling the same signs of fullness. This obviously highlights that persons with good cardiac awareness felt gastric signs of fullness more sensitively at a lower level of water intake and controlled water intake according to a more subtle recognition of visceral gastric signals compared to persons with poor cardiac awareness. This is in accordance with findings demonstrating that interoceptive awareness as measured by cardiac awareness is positively related to behavioral self-control according to a more sensitive perception of internal bodily signals such as cardiovascular changes and feelings of fatigue in a setting that allows persons to decide upon behavioral control of e.g. physical effort [13]. The drink test represents a situation where subjects can decide on the amount of water ingestion according to their subjective feelings of perceived fullness. According to these findings our results suggest that cardiac awareness is related to the self-control of water ingestion due to a better sensitivity for gastric signals of feeling fullness.

These results also speak in favor of the validity of the ingested amount of water during the WLT as an indicator for individual differences in gastric sensations of fullness or satiety and argues against the possibility that the amount of ingested water volume could potentially also reflect individual differences in further possibly intervening variables, such as personal preferences for or against water or different feelings of nausea that could have influenced the WLT procedure. Moreover, there was no relationship neither between anxiety measures and heartbeat perception nor anxiety indicators and ingested water volume underscoring that results were not confounded by anxiety.

This interpretation is corroborated by the EGG data showing that, while there were no associations between cardiac awareness and EGG activity during baseline, there was a significant negative association between %3 cpm increase and heartbeat perception. Furthermore, good compared to poor heartbeat perceivers reacted with less %3 cpm increase during WLT. In conjunction with the findings outlined above showing that cardiac awareness is associated with a less amount of water ingestion according to a more sensitive perception of gastric signals of fullness, the results that cardiac awareness is associated with less %3 cpm reactivity are in line with expectations. As in healthy subjects the volume required to produce fullness in a drink test has been demonstrated to correspond to an increase of 3 cpm activity of the EGG [35], good heartbeat perceivers ingesting less water volume in the drink test should show less increase in normogastria. The latter underscores that greater sensitivity for gastric signals is not dependent on greater gastric reactivity or “gastric signal intensity” in response to a gastric stimulus such as water intake, but is primarily based on the more sensitive perception of gastric cues.

In summary our results demonstrate that there is significant overlap or coherence of interoceptive awareness across different visceral modalities regarding cardiac and gastric signals, which both represent bodily cues that also show perceivable changes during situations of daily life (e.g. changes of heart rate during stress or arousal, states of hunger or satiety).

These findings are in line with the characterization of interoception representing the sense of the physiological condition of the entire body and of different bodily signals with the anterior insula as a relevant brain site for the integration of homeostatic states of different bodily tissues with motivational and emotional processes, supporting feeling states and giving rise to conscious visceral perception [21]–[22], [73]. There is relevant evidence supporting the role of the anterior insula for cardiac awareness [6], [30] as well as for interoceptive stimuli arising from the stomach, oesophagus and rectum [22]–[27]. Furthermore, anatomical evidence demonstrated that interoceptive afferent fibers of the lamina I spinothalamocortical pathway project via brainstem nuclei and specific thalamic nuclei to the dorsal posterior insula bilaterally [21], monitoring the physiological state of all internal bodily signals [21].

At this point, one crucial aspect has to be discussed which is of interest for future studies investigating the neural correlates of interoceptive awareness across different modalities. It has been proposed [20]–[22] that interoceptive afferent inputs provide somatotopically organized cortical representations of the physiological condition of the body and converge via an iterative posterior to anterior re-mapping within the insula in order to give rise to a progressive incorporation with multimodal information regarding emotionally salient environmental stimuli and support a constellation of motivationally significant bodily sensations [21], [22]. This model [21]–[22], [73], [74] allows the conclusion that there are somatotopical representations for different visceral inputs of the body that could be the basis for different re-representations and integrations into and within the anterior insula for different organ systems depending on the kind of visceral input. According to this logic different individual “sensitivities” for different organ systems, probably in association with different feelings states and with possibly different somatotopic segregations also within the anterior insula [75], should also be possible.

On the basis of this assumption, and in the context of results showing greater activity of the anterior insula cortex in persons with greater cardiac awareness [6], [30], our results could suggest that the observed association between trait-like interoceptive cardiac awareness and sensitivity for gastric signals is also closely linked anatomically and functionally within a neural network of subsets of visceral representations in the anterior insula cortex. Thus, our findings encourage future studies investigating the activity, morphology and connectivity of relevant brain regions during different interoceptive tasks under comparable conditions and with comparable methods, accounting for habitual differences in interoceptive awareness.

Furthermore, recent results demonstrated that cardiac awareness is positively associated with benefits in selective and divided attention [12] suggesting greater cardiac awareness to represent an indicator of self-focused attention. It could be proposed that persons with greater sensitivity to cardiac signals are also more able to focus attention to further bodily signals such as gastric signals depending on the demands of the specific task or an equivalent situation of life. According to this interpretation, a better attentive “focus on the body” during different situations could be of relevance for the association between cardiac awareness and gastric sensitivity. Likewise, recent data demonstrated that ventral and anterior regions of the insula representing relevant “interoceptive sites” also support processes related to focal attention [76]–[77].

Finally, our findings are the first to demonstrate a congruency between the sensitivity for cardiac and gastric signals without using invasive methods that are limited by potentially influencing and manipulating sensitivity of different bodily modalities. “Interoceptive awareness” as assessed by heartbeat perception seems to represent a better ability to focus, to perceive and to process internal bodily information across visceral modalities, such as gastric signals, with cardiac and gastric signals both representing bodily cues that show perceivable activity changes during situations of everyday life. Future studies are encouraged, combining brain imaging and measures of interoceptive awareness, in order to elucidate the role of the anterior insula regarding somatotopic re-representations of different visceral signals lending support to interoception.
